# Transcriptional Activity of Human Epidermal Growth Factor Receptor Family and Angiogenesis Effectors in Locoregionally Recurrent Head and Neck Squamous Cell Carcinoma and Correlation with Patient Outcome

**DOI:** 10.1155/2009/854127

**Published:** 2009-10-07

**Authors:** George Pentheroudakis, Nikolaos Angouridakis, Ralph Wirtz, Angelos Nikolaou, Konstantine T. Kalogeras, Nicholas Pavlidis, George Fountzilas

**Affiliations:** ^1^Department of Medical Oncology, Ioannina University Hospital, Ioannina 45500, Greece; ^2^ENT Department, “AHEPA” Hospital, Aristotle University of Thessaloniki School of Medicine, Thessaloniki 57100, Greece; ^3^Siemens Healthcare Diagnostics, Cologne, Germany; ^4^Hellenic Cooperative Oncology Group, Data Office, Athens 45118, Greece; ^5^Department of Medical Oncology, “Papageorgiou” Hospital, Aristotle University of Thessaloniki School of Medicine, Thessaloniki 57100, Greece

## Abstract

Locoregional recurrence is the most common failure pattern in patients with head and neck squamous cell carcinoma (HNSCC). We retrospectively identified 41 HNSCC patients with locoregional relapse and used kinetic reverse transcription-polymerase chain reaction (kRT-PCR) in order to study fresh-frozen tumour messenger RNA (mRNA) levels of the Human Epidermal growth factor family members HER1-4, the Vascular Endothelial Growth Factors (VEGFs) A, B, C, D, and their receptors VEGFR1, 2, 3. High VEGF-C and VEGFR3 tumour mRNA expression correlated with relapse beyond the primary locus (neck nodes or soft tissues, *P* < .05). Tumours with regional nodal involvement at diagnosis more often exhibited high transcriptional activity of VEGFR1 and VEGFR3 at the time of relapse (*P* < .05). At a median follow-up of 52 months from the time of locoregional recurrence, patients with high VEGF-C tumours at relapse had significantly poorer postrelapse progression-free survival (R-PFS, 5 versus 47 months, log-rank *P* = .052) and a trend for inferior postrelapse overall survival (R-OS, 22 versus 44 months, log-rank *P* = .076) in comparison to low VEGF-C tumours. Similar association with dismal outcome was seen for its receptor, VEGFR3 tumoural mRNA levels (log-rank *P* = .060). In contrast, suppressed tumour transcription of VEGF-D was associated with poorer post-relapse survival, though statistical significance was not reached. Active transcription of the VEGF-C/VEGFR3 axis in recurrent HNSCC is associated with failure at neck soft tissues/lymph nodes and inferior survival post-relapse.

## 1. Introduction

Locoregional recurrence is the most common pattern of failure after definitive treatment of head and neck squamous cell carcinoma (HNSCC), despite increasing use of combined modality approaches incorporating chemotherapy, radiotherapy, and surgery as initial management of patients with locally advanced tumours [1]. Failure to achieve control of locoregional disease increases the likelihood of distant metastases and compromises patient survival and quality of life. Even in patients succumbing to distant metastatic disease, uncontrolled cancer at the primary site or neck is seen in 90% of the cases [2]. In several large series and multi-institutional trials, the rate of locoregional relapse ranged from 20% to 57%, the most important predictors for failure being involved resection margins, regional nodal metastases, advanced T stage, high grade, neurogenic/vessel invasion, and p53 gene mutations [3]. In the occurrence of isolated locoregional recurrence, long-term disease control is achieved in a minority of patients (10%–25%), namely, those able to undergo surgical salvage and/or re-irradiation. Clinicopathological parameters that predict outcome of patients with HNSCC locoregional recurrence have been reported in a number of studies and included time interval from diagnosis to relapse, bulk, site, and resectability of recurrence, ability to re-irradiate at doses >60 Gy, and performance status [4, 5]. However, no data are available on molecular tumour biomarkers of potential prognostic/predictive significance for the outcome of patients with locoregionally recurrent HNSCC. Several investigators have reported overexpression of Human Epidermal growth factor Receptor (HER) family members and active angiogenic activity in HNSCC, with important implications since therapeutic compounds targeting these cellular pathways are available. In view of the above, we studied the tumour transcriptional activity of HER and vascular endothelial growth factor (VEGF/VEGFR) pathways at the occurrence of locoregional recurrence, retrospectively examined associations with clinicopathological characteristics and analyzed their utility for predicting patient outcome following relapse.

## 2. Patients and Methods

Patients with localized stage I-III HNSCC managed between January 2002 and August 2004 at the ENT Department of the Aristotle University of Thessaloniki with potentially curative surgery and/or radical external beam irradiation and subsequently experiencing isolated locoregional recurrence were retrospectively identified. Isolated locoregional recurrence was defined as one occurring in the primary site, neck nodes or neck soft tissues in the absence of distant metastases. This constituted the criterion for patient identification and for the study of HER/VEGF pathways in fresh tumour tissue biopsies obtained at the time of locoregional recurrence and snap-frozen at −80°C . A waiver of consent for the use of biologic material was provided by the Bioethics Committee of the Aristotle University of Thessaloniki.

Intact RNA of high quality as determined by analysis of the housekeeping gene RPL37A was isolated from 41 fresh-frozen tumour tissue samples with tumour cellularity of at least 70%. Approximately 50 mg of fresh-frozen tumor tissue were crushed in liquid nitrogen. RLT-Buffer (QIAGEN, Hilden, Germany) was added and the homogenate was centrifuged through a QIAshredder column (QIAGEN). From the eluate, total RNA was isolated using the RNeasy Kit (QIAGEN) according to the manufacturer's instructions. RNA yield was determined by UV absorbance, and RNA quality was assessed by analysis of ribosomal RNA band integrity on an Agilent 2100 Bioanalyzer RNA 6000 LabChip kit (Agilent Technologies, Palo Alto, CA). Kinetic reverse transcription-polymerase chain reaction (kRT-PCR) was applied for the assessment of messenger RNA (mRNA) expression of HER1 (EGFR), HER2, HER3, HER4, VEGF-A (all isoforms), VEGF-B, VEGF-C, VEGF-D, VEGFR1 (FLT1), VEGFR2 (KDR), and VEGFR3 (FLT4) using the following TaqMan-based primer/probe sets:

 VEGF-A Probe CACCATGCAGATTATGCGGATCAAACCT

 Forward Primer GCCCACTGAGGAGTCCAACA

 Reverse Primer TCCTATGTGCTGGCCTTGGT

 VEGF-B Probe CACATCTATCCATGACACCACTTTCCTCTGG

 Forward Primer TGGCAGGTAGCGCGAGTAT

 Reverse Primer CCCTGTCTCCCAGCCTGAT

 VEGF-C Probe TTGAGTCATCTCCAGCATCCGAGGAAA

 Forward Primer CCACAGATGTCATGGAATCCAT

 Reverse Primer TGCCTGGCTCAGGAAGATTT

 VEGF-D Probe TGACATTGAAACACTAAAAGTTATAGATGAAGAATGGCA

 Forward Primer ACTAGGTTTGCGGCAACTTTCT

 Reverse Primer TCTCTAGGGCTGCACTGAGTTCT

 FLT1 Probe TGCTGTCGCCCTGGTAGTCATCAAACA

 Forward Primer CATGGGAGAGGCCAACAGA

 Reverse Primer AACCTTTGAAGAACTTTTACCGAATG

 KDR Probe TCTTGGCATCGCGAAAGTGTATCCACA

 Forward Primer TTCCAAGTGGCTAAGGGCAT

 Reverse Primer CGTGCCGCCAGGTCC

 FLT4 Probe TGCCTGCTTCCCTGGGTAGTCCC

 Forward Primer GCACCCACTTACCCCGC

 Reverse Primer GAGTTTAACTCAGGTGTCACCTTTGA

Forty cycles of amplification were applied, and the cycle threshold (CT) values of the target genes were identified. CT values were normalized by subtracting the CT value of the housekeeping gene RPL37A from the CT value of the target gene (ΔCT). RNA results were then reported as 40-ΔCT values, which would correlate proportionally to the mRNA expression level of the target gene. Human reference total RNA pooled from ten human cell lines (Stratagene, La Jolla, CA) was used as a positive control. RNA-free DNA extracted from tumor tissues was used as a negative control.

We sought to study the distribution of biomarker values, the correlation of biomarkers to various clinicopathological parameters at first diagnosis and at the time of recurrence, the association of biomarkers with time from diagnosis to relapse (relapse-free interval, RFI), and their predictive significance for relapse-related progression-free survival (R-PFS) and overall survival (R-OS). RFI was measured from initial diagnosis until the time of isolated locoregional recurrence, R-PFS from the time of isolated locoregional relapse until verified disease progression, and death or last contact and R-OS from locoregional relapse until death from any cause or date of last contact. Disease progression (R-PFS event) was considered to be an increase in tumour maximal diameter of >20% or appearance of new lesions despite salvage therapy. Both R-OS and R-PFS were estimated using the Kaplan-Meier product-limit method, and comparisons were performed using the log-rank test. 

Categorical data were presented as counts and corresponding percentages, while the continuous variables were summarized using the medians and ranges. Distributional studies of gene mRNA expression values confirmed the absence of natural cut-offs in frequency histograms, while the small sample size further supported the use of the median as the optimal cut-off. Gene mRNA expression was considered low or negative when below the median of all measured mRNA values and high or positive when above the median and was used as a categorical variable in the analysis. Comparisons between mRNA expression and categorical variables were performed using the Fisher's exact test. The level of significance for all statistical tests was *α* = 0.05. Analysis was conducted using the SPPS for Windows, version 15.

## 3. Results

### 3.1. Clinicopathological Characteristics

Forty-one male patients, mostly heavy smokers and consumers of alcohol, initially presented at a median age of 65 with hoarseness and dysphagia. Diagnostic work-up led to diagnosis of squamous cell carcinoma of the larynx predominantly (90% of cases), mostly stage T1-3 (88% of cases), more often node-negative (85%), and moderately-well to well differentiated (61%). Initial management consisted of surgical resection of the tumour by either local excision (24%), segmental (19%), or total (24%) laryngectomy, whereas in one-third of the cases only a bioptic procedure was done and radical external beam radiotherapy was administered. Adjuvant chemotherapy was not administered, with the exception of one patient. Locoregional relapse occurred after a median of 15 months in the primary site (66%), neck lymph nodes (15%), or neck soft tissues (19%) and was managed by means of surgical resection (65% of patients) and/or irradiation (24%) and chemotherapy (24%). At the time of relapse, 46% of patients were managed with surgery only and 19% with resection followed by irradiation or chemotherapy. Among the 24% of patients who received radiotherapy at relapse, 17% had external beam radiotherapy only and 7% concurrent chemoradiation. No patients received re-irradiation. Among five patients who had chemotherapy administered and available data, three were treated with paclitaxel/liposomal doxorubicin, one with paclitaxel/gemcitabine, and one with weekly methotrexate. Clinicopathological characteristics at first diagnosis and at locoregional relapse are summarised in Table 1.

### 3.2. Association of Biomarkers with Clinicopathological Parameters

High versus low mRNA expression of HER1-4 genes, VEGF-A, B, C, D genes, and receptors VEGFR1, R2, R3 were examined for associations with alcohol consumption, tobacco consumption, age, and nodal status at initial diagnosis, relapse-free interval, site, size, and grade at relapse.

High VEGF-C transcription correlated significantly with tropism for relapse beyond the primary site: 50% of relapsing patients with high tumoural VEGF-C mRNA expression relapsed in lymph nodes or soft tissues versus only 15% of those who harboured tumours with low VEGF-C (test, *P* = .009). The same association was observed for tumoural transcription of VEGFR3 and the receptor of VEGF-C: tumours with high mRNA expression of VEGFR3 relapsed in neck nodes or soft tissues in 53% of the recurrent cases, while those with low expression relapsed in only 10% (*P* = .017). Tumoural VEGF-C/VEGFR3 mRNA expression may be a marker of predilection for relapse in regional lymph nodes/soft tissues rather than the primary site.

Tumours with regional nodal involvement at diagnosis more often exhibited high transcriptional activity of VEGFR1 or VEGFR3 at the time of relapse (test, *P* < .05). Among tumours profiled with high mRNA expression of VEGFR1 or VEGFR3 at relapse, regional nodal involvement had occurred in approximately 20% of the cases at initial presentation. In sharp contrast, no nodal metastases had been present at initial diagnosis in cases where tumoural VEGFR1 or VEGFR3 mRNA expression at relapse was low. This preliminary finding deserves further investigation, as it appears that profiling of VEGFR1 and VEGFR3 in HNSCC patients at initial diagnosis may be of potential value for predicting nodal involvement or locoregional relapse.

In addition, a trend was found for high VEGFR1 tumoural expression at relapse to be associated with tropism for nodal or soft tissue failure (test, *P* = .056), and for high VEGF-B with a history of high alcohol consumption (*P* = .075). No other clinically or statistically significant associations of studied biomarkers with clinicopathological characteristics were seen. Table 2 summarizes the biomarkers with the most significant associations with clinicopathological data, while all associations of the HER family genes with clinicopathological data are shown in Table 3.

### 3.3. Predictive Significance for RFI

Transcriptional activity of any of the studied biomarkers was not significantly associated with occurrence of early or late locoregional relapse (RFI of less versus more than 12 months). Moreover, transcription of the studied biomarkers could not predict the timing of relapse, even when the latter was examined as a continuous time variable (Mann-Whitney U test, *P* > 0.1).

### 3.4. Predictive Significance for R-PFS

**Figure 1:**
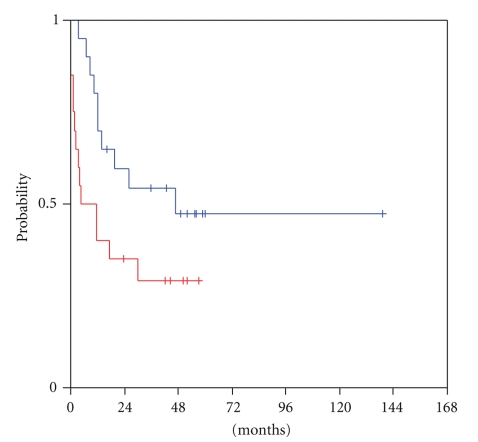
Relapse-related PFS in patients with low (blue line) and high (red line) tumour VEGF-C mRNA expression.

**Figure 2:**
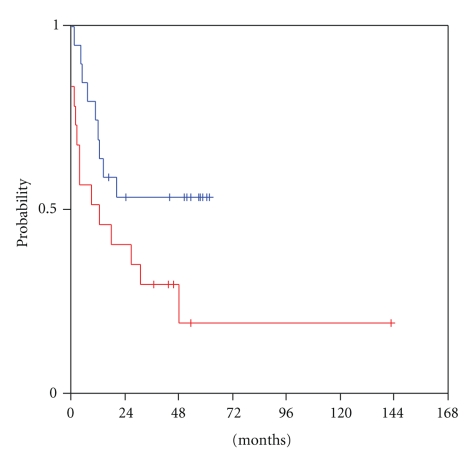
Relapse-related PFS in patients with low (blue line) and high (red line) tumour VEGFR3 mRNA expression.

**Figure 3:**
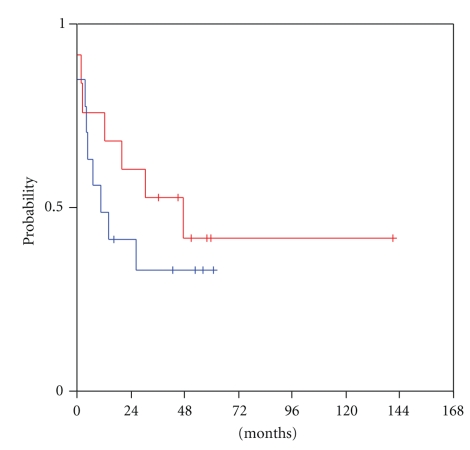
Relapse-related PFS in patients with low (blue line) and high (red line) tumour VEGF-D mRNA expression.

At a median follow-up of 52 months from the time of locoregional recurrence (range 8–53 months), transcriptional activity of HER and VEGF/VEGFR family members was examined for predictive significance for survival from relapse until progression or death (R-PFS). High mRNA expression of VEGF-C in the tumour at the time of locoregional recurrence was significantly associated with shorter progression-free survival (log-rank, *P* = .052). Patients who harboured tumours with low VEGF-C mRNA expression had a median R-PFS of 47 months versus a median R-PFS of only 5 months for the patients with tumours expressing high VEGF-C (Figure 1). Moreover, mRNA expression levels of its receptor, VEGFR3, were related to patient outcome with a trend for statistical significance (log-rank, *P* = .060). Patients with high tumour transcription of VEGFR3 at relapse reached a median R-PFS of only 12 months, in contrast to those harbouring tumours with low VEGFR3 mRNA expression, in whom the median R-PFS had not been reached yet at the time of the analysis (Figure 2). An association of tumour VEGF-D expression and R-PFS was speculated, though no statistical significance was observed (log-rank, *P* = 0.41). Low tumour VEGF-D mRNA expression was associated with a median R-PFS of only 10 months, while high VEGF-D with a median R-PFS of 47 months (Figure 3).

### 3.5. Predictive Significance for R-OS

**Figure 4:**
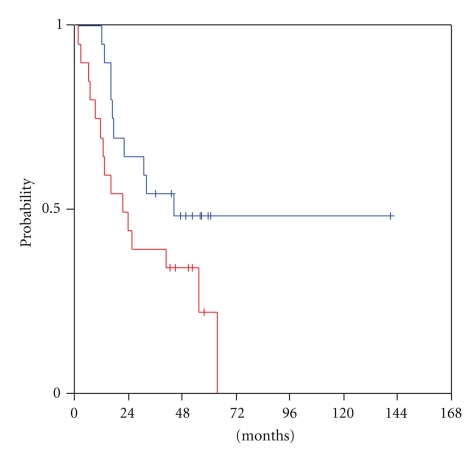
Relapse-related OS in patients with low (blue line) and high (red line) tumour VEGF-C mRNA expression.

**Figure 5:**
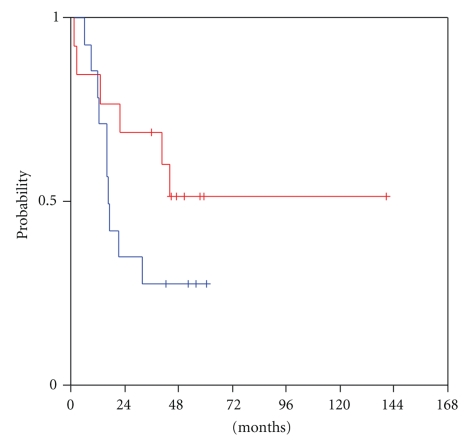
Relapse-related OS in patients with low (blue line) and high (red line) tumour VEGF-D mRNA expression.

Among all studied biomarkers, only VEGF-C tumoural transcription at recurrence exhibited a trend for a statistically significant association with survival of relapsed patients (log-rank, *P* = .076). Those patients who harboured tumours with high VEGF-C at relapse had a median R-OS of 22 months, whereas patients with low-level tumour VEGF-C had a median survival of 44 months (Figure 4). Of note, high tumour expression levels of VEGF-D at locoregional recurrence were associated with an improved patient outcome, albeit not statistically significant (log-rank, *P* = .15), as had been the case with R-PFS. In cases with low tumour VEGF-D levels, the median R-OS was only 17 months, in contrast to cases with high VEGF-D tumour mRNA expression, in which the median survival had not been reached yet, at a median follow-up of 52 months (Figure 5).

## 4. Discussion

The impact of locoregional recurrence in patients with HNSCC is devastating in several aspects: function, cosmesis, quality of life, and most importantly, survival. Standard clinical and pathological factors of established prognostic significance for patient outcome have been reported: resection margins, regional nodal metastases, advanced T stage, high grade, and neurogenic/vessel invasion [6, 7]. Still, 20%–30% of the patients with localised T1-T2 disease managed with negative margin resection, nodal clearance, and postsurgery irradiation eventually recur in the neck [1, 2]. EGFR (HER1), HER2, HER3, and HER4 transmembrane receptors are essential for proliferation, motility, and invasion of the malignant cell, with the former two having been studied more extensively. The rate of HNSCC tumours presenting immunohistochemical (IHC) protein overexpression was found to be 80%–90% for EGFR and 4%–39% for HER2 [8, 9]. Although EGFR and HER2 IHC protein expression was shown to be of prognostic value for inferior clinical outcome, they were unreliable predictors of benefit from targeted therapeutic agents [10]. Especially EGFR is expressed in almost all HNSCC tumours, in keeping with the squamous cell phenotype, while its immunohistochemical protein staining is a subjective assay lacking the dynamic range of quantitative evaluation. EGFR and other HER family members form heterodimers upon ligand binding and activate intracellular signalling cascades that regulate survival, proliferation, motility, and angiogenesis of the malignant cell cluster. Recent large phase III trials showed overall survival benefit from the combination of the anti-EGFR monoclonal antibody cetuximab with radiotherapy or chemotherapy in patients with locally advanced or metastatic HNSCC [11, 12]. This clinical breakthrough makes imperative the need for the identification of biomarkers that would predict tumour response or resistance to EGFR-modulating agents.

VEGF protein overexpression assessed by IHC was found in 90% of HNSCC tumours, associated with a 2-fold higher risk of death at two years [13]. The five VEGF ligands (VEGF-A, B, C, D, and E) interact as dimers with the three types of VEGF receptors (VEGFR1, 2 and 3) found on endothelial and tumour cells. Receptor homo- or heterodimerisation initiates complex intracellular signalling mechanisms leading to formation of new tumour blood vessels (VEGFR1 and 2) or lymph vessels (VEGFR3) [14]. Therapeutic agents targeting the VEGF ligands or receptors inhibit neoplastic angiogenesis, optimise remaining vasculature, decrease interstitial fluid pressure, and synergistically kill tumour cells when given in combination with chemotherapy or radiotherapy in preclinical models [15]. Bevacizumab, a monoclonal antibody that binds VEGF, and tyrosine kinase inhibitors of the VEGF receptors are currently being evaluated in HNSCC patients, along with biomarkers that could predict for benefit from such targeted therapies. Seiwert et al. recently reported that the ratio of phosphorylated VEGFR2 to total VEGFR2, measured by immunofluorescence, predicts for response in patients with recurrent or metastatic HNSCC receiving bevacizumab/erlotinib combination therapy [16].

Gene transcriptional profiling of messenger RNA by means of real time kRT-PCR provides a quantitative evaluation method that is not affected by observer variability or the widely known IHC technique limitations. In order to screen for molecular predictors of outcome of patients with recurrent HNSCC, we studied fresh-frozen tumours from 41 patients with locoregional recurrence of relatively low-risk disease at presentation: the median tumour size was 2 cm, 68% of cases being T1-2, 85% N0, and 61% well to moderately well differentiated. Despite the small sample size, transcriptional activation of the VEGF-C/VEGFR3 axis at relapse was associated with recurrence outside the primary site (neck nodes or soft tissues) and inferior progression-free and overall survival from relapse at a marginal statistical significance. Moreover, tumours that were node-positive at presentation had higher VEGFR1 and VEGFR3 mRNA expression levels at relapse. Despite the preliminary nature of these findings, in a small retrospective cohort, the emergence of statistically significant associations of angiogenesis effectors with outcome, in patients initially presenting with low-risk tumours, hints for the presence of clinical significance and a more robust correlation, should the sample size had been larger.

Our observation incriminating tumoural VEGF-C/VEGFR3 signalling in nodal/soft tissue relapse and poor post-relapse outcome is in keeping with recent published evidence. Several investigators reported association of protein or mRNA expression of VEGF-C with lymphatic metastases and invasion in HNSCC, gastric, prostate, and breast cancer cell lines and small patient series [17–19]. Of note, Tanigaki et al. found tumoural VEGF-A/VEGFR1 and 2 transcription correlated to development of distant metastases, while VEGF-C/VEGFR3 to locoregional recurrence [20]. Moreover, O-Charoenrat et al. reported that in contrast to other VEGF ligands, VEGF-D mRNA was suppressed in HNSCC tumours. Preclinical data have shown that active HER1/2 signalling upregulates VEGF-A and C and downregulates VEGF-D transcription in lung adenocarcinoma and HNSCC cell lines [21]. We observed inferior R-PFS and R-OS in patients harbouring tumours with low VEGF-D mRNA expression compared to those with high VEGF-D, though statistical significance was not reached. VEGF-D may exert an antagonistic effect on neoplastic neovascularisation, forming heterodimers with VEGF-A, B, and C and modulating the activity of the VEGFR1, 2, and 3 along with the neuropilin receptors. This phenomenon of counter-regulation is probably extremely important for the fine-tuning of angiogenesis. Recently, VEGF-Ab, a splice variant of the powerful proangiogenic VEGF-A ligand, was shown to exert antiangiogenic effects in normal tissues and a variety of solid tumours [22].

The mechanism of the adverse prognostic impact of VEGF-C/VEGFR3 signalling may include dissemination of tumour cells in the systemic circulation and arrest in lymph nodes/distant sites, direct enhancement of lymph-angiogenesis, and creation of a permissive environment for tumour progression by the induction of adhesion molecules, growth factors, and proteolytic enzymes. In contrast to VEGF-C, HER signalling was not significant for predicting patient outcome, despite in vitro data emphasizing its key role in the control of cell cycle, invasion, and the induction of VEGF-A and C-mediated angiogenesis. Indeed, in an HNSCC patient series, protein expression of EGFR or HER2 could not predict benefit from chemotherapy or targeted therapies [8–11]. Only EGFR gene amplification activating EGFR gene mutations and the presence of the truncated form of the EGFRvIII protein correlated with clinical benefit or patient outcome [10]. Although our mRNA methodology could not screen for these biomarkers, Agulnik et al. found excess EGFR gene copy numbers in only 4 out of 37 patients, and Willmore-Payne et al. reported HER1/2 mutations in less than 10% of patients with HNSCC [23, 24]. In contrast, Chung et al. observed EGFR gene amplification in 58% of 75 recurrent or metastatic HNSCC patients and reported its association with poor outcome [25]. However, EGFR copy number status did not correlate with protein or mRNA expression. This could explain our inability to find any prognostic significance for EGFR mRNA levels in our study. Indeed, HER1/HER2 gene amplification may be an early oncogenic event, with most gene copies becoming transcriptionally inactive later. Alternate splicing of EGFR transcripts, not detected by our mRNA probes, could also offer another explanation [26]. Moreover, the EGFR/HER2 genes may carry prognostic information not associated with their amplification status per se but rather act as surrogate markers of genetic instability or of other coamplified genes [27]. Of note, Seiwert et al. reported that endothelial but not tumour cell EGFR protein levels correlated with response to bevacizumab + erlotinib [16]. Moreover, the combination reduced VEGFR2 and EGFR protein expression in neoplastic endothelia but not tumour cells. Our mRNA analysis, though based on frozen sections with ≥70% tumour cellularity, would not discriminate between tumour cells and neoplastic vessel endothelial cells. 

In conclusion, VEGF-C/VEGFR3 mRNA expression at relapse may be of potential value as a new biomarker predicting nodal/soft tissue regional relapse and poor outcome after recurrence in HNSCC patients. It should be stressed that molecular profiling of primary tumours is necessary in order to obtain prognostic information at diagnosis. Comparison of the molecular profiles of primary and matched recurrent tumors is required to derive safe conclusions and was not done in our study. Still, our findings may serve as hypothesis-generating data and, if validated in larger prospective series, may justify more aggressive neck management at presentation and treatment of HNSCC patients exhibiting high VEGF-C mRNA expression with targeted therapies (anti-VEGF-C antibodies, VEGFR3 tyrosine kinase inhibitors), either upfront or at recurrence, in order to optimise their outcome.

## Figures and Tables

**Table 1:** Clinicopathological characteristics at initial diagnosis and locoregional relapse.

	*N* = 41			
	At diagnosis		At recurrence	
Age				
Median (range)	65 (45–77)			
Relapse-free interval (months)				
Median (range)			15 (5–221)	
Size (cm)				
Median (range)	2 (0.3–6)		2.6 (0.6–10)	

	*N*	%	*N*	%
Gender				
Male	41	100		
Family history				
No	29	71		
Yes	12	29		
Smoking history				
No	2	5	28	68
Yes	39	95	13	32
Pack years				
Median (range)	52.5 (0–125)			
Alcohol consumption				
Low	13	32		
Moderate	16	39		
High	12	29		
Symptoms				
Hoarseness	26	63		
Dyshphagia	10	24		
Dyspnoea	1	2		
Sore mouth	2	5		
Ulceration	1	2		
Lymphadenopathy	1	2		
Primary site				
Glottic	26	63		
Supraglottic	10	24		
Transglottic	1	2		
Oropharynx	3	7		
Unknown primary	1	2		
Site of recurrence				
Local			27	66
Lymph nodes ± local			6	15
Other			8	19
T stage				
T1	16	39		
T2	12	29		
T3	8	20		
T4	4	10		
Unknown	1	2		
N stage				
N0	35	85		
N1	3	7		
N2	1	2		
Unknown	2	5		
Grade				
I	7	17	9	22
II	18	44	16	39
III	5	12	7	17
IV	1	2	2	5
In Situ	1	2	0	0
Verrucous	1	2	1	2
Unknown	8	20	6	15
Surgery				
Biopsy	13	32	14	34
Total laryngectomy ± nodal resection	10	24	19	46
Hemilaryngectomy or segmental resection	8	19	1	2
Local resection	10	24	7	17
Radiotherapy (RT)				
No	20	49	30	73
Yes	21	51	10	24
Unknown	0	0	1	2
RT dose (Gy)				
Median (range)	66 (64–74)		69 (40–72)	
Chemotherapy (CT)				
No	40	98	30	73
Yes	1	2	10	24
Unknown	0	0	1	2
CT duration (months)				
Median (range)			3.7 (1.8–5.0)	
Radiotherapy only	20	49	7	17
Chemotherapy only	0	0	7	17
Paclitaxel + gemcitabine			1	
Paclitaxel + liposomal doxorubicin			3	
Methotrexate			1	
Missing data			2	
Surgery only	19	46	19	46
Chemoradiotherapy	1	2	3	7
Paclitaxel + gemcitabine			1	
Paclitaxel + liposomal doxorubicin			1	
Missing data			1	

**Table 2:** Association of VEGF-C, VEGFR1 (FLT1), and VEGFR3 (FLT4) mRNA expression with clinicopathological parameters.

	VEGF-C			VEGFR1 (FLT1)			VEGFR3 (FLT4)		
	Low	High	*P*	Low	High	*P*	Low	High	*P*
Alcohol consumption			.168			.324			.999
Low	4 (20)	9 (45)		8 (40)	5 (25)		7 (35)	6 (32)	
Moderate	10 (50)	5 (25)		5 (25)	10 (50)		8 (40)	7 (37)	
High	6 (30)	6 (30)		7 (35)	5 (25)		5 (25)	6 (32)	
Site of relapse			**.009**			.056			**.017**
Local only	17 (85)	10 (50)		17 (85)	10 (50)		18 (90)	9 (47)	
Lymph nodes ± Local	3 (19)	3 (15)		2 (10)	4 (20)		1 (5)	5 (26)	
Other	0 (0)	7 (35)		1 (5)	6 (30)		1 (5)	5 (26)	
Size at 1st relapse			.712			.110			.999
<2 cm	3 (15)	4 (20)		6 (30)	1 (5)		4 (20)	3 (16)	
2–4 cm	10 (50)	9 (45)		11 (55)	10 (50)		11 (55)	9 (47)	
>4 cm	5 (25)	2 (10)		2 (10)	5 (25)		4 (20)	3 (16)	
Unknown	2(10)	5 (25)		1 (5)	4 (20)		1 (5)	4 (21)	
Lymph nodes at diagnosis			.342			**.047**			**.041**
N0	19 (95)	16 (80)		20 (100)	15 (75)		20 (100)	14 (74)	
N1-N2	1 (5)	3 (15)		0 (0)	4 (20)		0 (0)	4 (21)	
Unknown	0 (0)	1 (5)		0 (0)	1 (5)		0 (0)	1 (5)	
Differentiation grade at relapse			.697			.697			.697
Well or moderate	14 (70)	10 (50)		14 (70)	10 (50)		14 (70)	10 (53)	
Poor or undifferentiated	4 (20	5 (25)		4 (20)	5 (25)		4 (20)	5 (26)	
Unknown	2 (10)	5 (25)		2 (10)	5 (25)		2 (10)	4 (21)	
Pack years exposure			.341			.341			.751
<52.5	9 (45)	13 (65)		9 (45)	13 (65)		10 (50)	11 (58)	
>52.5	11 (55)	7 (35)		11 (55)	7 (35)		10 (50)	8 (42)	
Age			.527			.999			.999
<65	9 (45)	12 (60)		11 (55)	10 (50)		10 (50)	10 (53)	
>65	11 (55)	8 (40)		9 (45)	10 (50)		10 (50)	9 (47)	
Diagnosis to recurrence interval			.333			.748			.748
<12 months	6 (30)	10 (50)		7 (35)	9 (45)		9 (45)	7 (37)	
>12 months	14 (70)	10 (50)		13 (65)	11 (55)		11 (55)	12 (63)	

**Table 3:** Association of mRNA expression the HER family genes with clinicopathological parameters.

	EGFR			HER2			HER3			HER4		
	Low	High	*P*	Low	High	*P*	Low	High	*P*	Low	High	*P*
	*N* = 21	*N* = 20		*N* = 18	*N* = 18		*N* = 21	*N* = 20		*N* = 20	*N* = 19	
Alcohol Consumption			.577			.404			.259			.239
Low	5 (24)	8 (40)		8 (44)	5 (28)		9 (43)	4 (20)		9 (45)	4 (21)	
Moderate	9 (43)	7 (35)		7 (39)	6 (33)		6 (29)	10 (50)		6 (30)	10 (53)	
High	7 (33)	5 (25)		3 (17)	7 (39)		6 (29)	6 (30)		5 (25)	5 (26)	
Site of relapse			.999			.501			.812			.545
Local only	14 (67)	13 (65)		13 (72)	13 (72)		13 (62)	14 (70)		12 (60)	15 (79)	
Lymph nodes ± Local	3 (14)	3 (15)		2 (11)	4 (22)		4 (19)	2 (10)		3 (15)	2 (11)	
Other	4 (19)	4 (20)		3 (17)	1 (6)		4 (19)	4 (20)		5 (25)	2 (11)	
Size at 1st relapse			.425			.256			.145			.716
<2 cm	4 (19)	3 (15)		3 (17)	4 (22)		4 (19)	3 (15)		3 (15)	4 (21)	
2–4 cm	9 (43)	13 (65)		8 (44)	10 (56)		9 (43)	13 (66)		11 (55)	10 (53)	
>4 cm	5 (24)	2 (10)		5 (28)	1 (6)		6 (29)	1 (5)		5 (25)	2 (10)	
Unknown	3 (14)	2 (10)		2 (11)	3 (17)		2 (10)	3 (15)		1 (5)	3 (16)	
Lymph nodes at diagnosis			.999			.999			.999			.999
N0	18 (86)	18 (90)		16 (89)	16 (89)		18 (86)	18 (90)		17 (85)	17 (89)	
N1-N2	2 (10)	2 (10)		2 (11)	2 (11)		2 (10)	2 (10)		2 (10)	2 (11)	
Unknown	1 (5)	0 (0)		0 (0)	(0)		1 (5)	0 (0)		1 (5)	0 (0)	
Differentiation grade at relapse			.240			.417			.999			.448
Well or moderate	13 (62)	12 (60)		13 (72)	9 (50)		12 (57)	13 (65)		12 (60)	13 (81)	
Poor or undifferentiated	2 (10)	7 (35)		3 (17)	5 (28)		4 (19)	5 (25)		6 (33)	3 (19)	
Unknown	6 (29)	1 (5)		2 (11)	4 (22)		5 (24)	2 (10)		2 (10)	3 (16)	
Pack years exposure			.999			.999			.999			.205
<52.5	11 (52)	11 (55)		10 (56)	9 (50)		11 (52)	11 (55)		8 (40)	12 (63)	
>52.5	10 (48)	9 (45)		8 (44)	9 (50)		10 (48)	9 (45)		12 (60)	7 (37)	
Age			.217			.094			.538			.752
<65	13 (62)	8 (40)		12 (67)	6 (33)		12 (57)	9 (45)		11 (55)	9 (47)	
>65	8 (38)	12 (60)		6 (33)	12 (67)		9 (43)	11 (55)		9 (45)	10 (53)	
Diagnosis to recurrence Interval			.530			.305			.341			.748
<12 months	7 (33)	9 (45)		9 (50)	5 (28)		10 (48)	6 (30)		9 (45)	7 (37)	
>12 months	14 (67)	11 (55)		9 (50)	13 (72)		11 (52)	14 (70)		11 (55)	12 (63)	

## References

[1] Ganly I, Kaye SB (2000). Recurrent squamous-cell carcinoma of the head and neck: overview of current therapy and future prospects. *Annals of Oncology*.

[2] Kotwall C, Sako K, Razack MS, Rao U, Bakamjian V, Shedd DP (1987). Metastatic patterns in squamous cell cancer of the head and neck. *American Journal of Surgery*.

[3] Wong SJ, Machtay M, Li Y (2006). Locally recurrent, previously irradiated head and neck cancer: concurrent re-irradiation and chemotherapy, or chemotherapy alone?. *Journal of Clinical Oncology*.

[4] Salama JK, Vokes EE, Chmura SJ (2006). Long-term outcome of concurrent chemotherapy and reirradiation for recurrent and second primary head-and-neck squamous cell carcinoma. *International Journal of Radiation Oncology Biology Physics*.

[5] De Crevoisier R, Bourhis J, Domenge C (1998). Full-dose reirradiation for unresectable head and neck carcinoma: experience at the Gustave-Roussy Institute in a series of 169 patients. *Journal of Clinical Oncology*.

[6] Jones KR, Lodge-Rigal RD, Reddick RL, Tudor GE, Shockley WW (1992). Prognostic factors in the recurrence of stage I and II squamous cell cancer of the oral cavity. *Archives of Otolaryngology*.

[7] Peters LJ, Goepfert H, Ang KK (1993). Evaluation of the dose for postoperative radiation therapy of head and neck cancer: first report of a prospective randomized trial. *International Journal of Radiation Oncology Biology Physics*.

[8] Ang KK, Berkey BA, Tu X (2002). Impact of EGFR expression on survival and pattern of relapse in patients with advanced head and neck carcinoma. *Cancer Research*.

[9] Wei Q, Sheng L, Shui Y, Hu Q, Nordgren H, Carlsson J (2008). EGFR, HER2, and HER3 expression in laryngeal primary tumors and corresponding metastases. *Annals of Surgical Oncology*.

[10] Le Tourneau C, Siu LL (2008). Molecular-targeted therapies in the treatment of squamous cell carcinomas of the head and neck. *Current Opinion in Oncology*.

[11] Karamouzis MV, Grandis JR, Argiris A (2007). Therapies directed against EGFR in aerodigestive carcinomas. *Journal of the American Medical Association*.

[12] Vermorken J, Mesia R, Vega V (2007). Cetuximab extends survival of patients with recurrent or metastatic SCCHN when added to first line platinum based therapy: results of a randomised phase III study. *Journal of Clinical Oncology*.

[13] Kyzas PA, Cunha IW, Ioannidis JP (2005). Prognostic significance of vascular endothelial growth factor immunohistochemical expression in head and neck squamous cell carcinoma: a meta-analysis. *Clinical Cancer Research*.

[14] Seiwert TY, Cohen EEW (2008). Targeting angiogenesis in head and neck cancer. *Seminars in Oncology*.

[15] Bozec A, Sudaka A, Fischel J-L, Brunstein M-C, Etienne-Grimaldi M-C, Milano G (2008). Combined effects of bevacizumab with erlotinib and irradiation: a preclinical study on a head and neck cancer orthotopic model. *British Journal of Cancer*.

[16] Seiwert TY, Davis DW, Yan D, Mauer AM, Karrison T, Kozloff M (2007). pKDR/KDR ratio predicts response in a phase I/II pharmacodynamic study of erlotinib and bevacizumab for recurrent or metastatic head and neck cancer. *Journal of Clinical Oncology*.

[17] Homer JJ, Prentice MG, Cawkwell L, Birchall M, Greenman J, Stafford ND (2003). Angiogenesis and the expression of VEGF-A and C in squamous cell carcinoma of the piriform fossa. *Archives of Otolaryngology*.

[18] Nakazato T, Shingaki S, Kitamura N, Saito C, Kuwano R, Tachibana M (2006). Expression level of VEGF-C and A in cultured human oral squamous cell carcinoma correlates respectively with lymphatic metastases and angiogenesis when transplanted in nude mouse oral cavity. *Oncology Reports*.

[19] Shintani S, Li C, Ishikawa T, Mihara M, Nakashiro K , Hamakawa H (2004). Expression of VEGF A, B, C, and D in oral squamous cell carcinoma. *Oral Oncology*.

[20] Tanigaki Y, Nagashima Y, Kitamura Y, Matsuda H, Mikami Y, Tsukuda M (2004). The expression of VEGF-A, C and receptors 1 and 3: correlation with lymph node metastasis and prognosis in tongue squamous cell carcinoma. *International Journal of Molecular Medicine*.

[21] O-Charoenrat P, Rhys-Evans P, Eccles SA (2001). Expression of VEGF family members in head and neck squamous cell carcinoma correlates with lymph node metastasis. *Cancer*.

[22] Harper SJ, Bates DO (2008). VEGF-A splicing: the key to anti-angiogenic therapeutics?. *Nature Reviews Cancer*.

[23] Agulnik M, Cunha-Santos G, Hedley D (2007). Predictive and pharmacodynamic biomarker studies in tumor and skin tissue samples of patients with recurrent or metastatic squamous cell carcinoma of the head and neck treated with erlotinib. *Journal of Clinical Oncology*.

[24] Willmore-Payne C, Holden JA, Layfield LJ (2006). Detection of EGFR- and HER2-activating mutations in squamous cell carcinoma involving the head and neck. *Modern Pathology*.

[25] Chung CH, Ely K, McGavran L (2006). Increased EGFR gene copy number is associated with poor prognosis in head and neck squamous cell carcinomas. *Journal of Clinical Oncology*.

[26] Xu YH, Ishii S, Clark AJ  (1984). Human EGFR cDNA is homologous to a variety of RNAs overproduced in A431 carcinoma cells. *Nature*.

[27] Bergamo NA, Rogatto SR, Poli-Frederico RC (2000). Comparative genomic hybridisation analysis detects frequent over-representation of DNA sequences at 3q, 7p and 8q in head and neck carcinomas. *Cancer Genetics and Cytogenetics*.

